# Effects of Environmental Change on Travelers’ Sleep Health: Identifying Risk and Protective Factors

**DOI:** 10.3389/fpsyg.2020.00724

**Published:** 2020-05-13

**Authors:** Wei Xiong, Fang Fan, Haiying Qi

**Affiliations:** ^1^School of Psychology, South China Normal University, Guangzhou, China; ^2^School of Tourism Management, South China Normal University, Guangzhou, China; ^3^Key Laboratory of Brain, Cognition and Education Sciences, South China Normal University, Ministry of Education, Guangzhou, China; ^4^Center for Studies of Psychological Application, South China Normal University, Guangzhou, China; ^5^Guangdong Key Laboratory of Mental Health and Cognitive Science, South China Normal University, Guangzhou, China

**Keywords:** environmental change, sleep, insomnia, travelers, binary logistic regression

## Abstract

**Objective:**

The aim of this study is to predict the risk and protective factors for the differential effects of the environment on travelers’ sleep health.

**Methods:**

A sample of 505 travelers who stayed overnight in one of 28 hotels completed a sleep quality scale (SQS), a reduced scale of the Morningness/Eveningness questionnaire (rMEQ), and a hotel customer satisfaction questionnaire (H-SCI).

**Results:**

Individuals who are of morning type (*p* = 0.002), have reduced sleep duration (*p* = 0.010), and have high sensitivity to the sleep environment (*p* = 0.000) are most affected by environmental change. Interestingly, business travelers are more affected by sleep disturbances than leisure travelers, travelers who are more satisfied with hotels are less likely to experience insomnia in a new sleep environment (*p* = 0.000), and insomniacs are more likely to recover from insomnia during a trip (*p* = 0.000).

**Conclusion:**

Environmental change has an inconsistent impact on the sleep health of different individuals. In a new sleep environment, the possible risk factors for sleep health include (1) being of morning type, (2) reduced sleep duration during trip, (3) sensitivity to the sleep environment, and (4) business stress, while a possible protective factor is satisfaction with the hotel. Possible factors aiding in recovery from home insomnia include (1) being of evening type and (2) higher satisfaction with the hotel.

## Introduction

Mobility is one of the important characteristics of human activities in modern society (Meekan et al., 2017). In a very mobile society, people often experience different environments, and consequently experience disrupted and poor-quality sleep in these novel environments (Fischl et al., 1999). This effect is referred to as the first-night effect (FNE) in human sleep research and has been regarded as a typical sleep disturbance (Amzica and Steriade, 1997; Dale et al., 1999; Jahnke et al., 2012). Some research has demonstrated that when in a new environment, there is interhemispheric asymmetry in the regional slow-wave activity (SWA), and people display signs of vigilance and responsiveness as a protective measure (Tamaki et al., 2016). It is more likely that the origin of the FNE is multifactorial and includes several factors, but in most cases, studies have been performed in specialized sleep units, which adds yet another factor: change in environment (Le Bon et al., 2001). Furthermore, the FNE might last more than one night (Le Bon et al., 2001) and may even develop into acute insomnia. Therefore, when studying the onset of acute insomnia, the circumstances surrounding its triggering factors should also be accounted for Thomas et al. (2005); Ellis et al. (2012). Therefore, these studies have also shown that unfamiliar surroundings could be a potential cause of poor sleep, which can further develop into acute insomnia; however, surprisingly, some research has also demonstrated that insomniacs sleep better in novel places than in their homes (Hauri and Fisher, 1986), including hotels (Pallesen et al., 2016).

The stress sensitization model argues that individuals become increasingly sensitized to stress over time, so that the level of stress needed to trigger episode onsets becomes increasingly lower with each successive episode (Catherine, 2018). The model posits that in response to repeated exposure to both affective episodes and external stress, individuals become sensitized to stress, such that stressors from an unfamiliar sleep environment become increasingly relevant with successive bad sleep. In our research, the stress sensitization model supports the hypothesis that the mechanism underlying the impact of stress on bad sleep is explained through environmental changes. Accordingly, in an attempt to explain why some individuals develop conditioned reinforcement of insomnia when their sleep environment changes whereas others do not or sleep better while traveling than they do when at home, we recruited hotel customers who had experienced an overnight stay and measured their sleep change pattern, as well as possible risk and protective factors.

Hotels are an excellent site for sleep research because they are generally associated with environmental change due to their primary use in travel. The principle function of a hotel is to provide a place for people to sleep, and the decision to book a room in a hotel is informed by the likelihood of having a good night’s sleep (Lockyer and Roberts, 2009). Furthermore, hotels are architecturally designed in such a way that they are dedicated to sleep and restful activities (Valtonen and Veijola, 2011) and encourage sleep (Kraftl and Horton, 2008).

To our knowledge, there is some research that has investigated the relationship between sleep and hotels (Pallesen et al., 2016; Chen et al., 2018; Mao et al., 2018). For example, Mao et al. (2018) confirmed that the attributes of hotels, as well as the characteristics of trips, positively influence the quality of tourists’ sleep. In their research, however, Mao et al. did not gain greater insight into individual physical and psychological aspects. In fact, a great deal of research into mental health has shown that individuals’ sleep habits and their emotional state impact their sleep quality in their daily lives (Selvi et al., 2010; Gruber and Cassoff, 2014), but research has neglected to explore to what extent these personal characteristics will influence sleep health in a new sleep environment. What is known, however, is that tourists’ sleep quality positively affects their reviews of hotels, with good sleep quality resulting in high customer satisfaction (Chen et al., 2018; Radojevic et al., 2018). What remains unknown is whether tourists’ satisfaction with the hotels where they sleep also has an impact on travelers’ sleep change compared with sleep at home.

Therefore, the aim of the current study is to determine how environmental changes predict whether individuals will develop travel-related insomnia. The specific study aims are: (1) explore the overall changes in people’s experience on sleeping in an unfamiliar environment, (2) identify factors that increase the risk of developing travel insomnia, (3) ascertain factors that protect people from developing travel insomnia, and (4) identify factors that assist in the recovery of home insomniacs sleeping in a new environment.

## Materials and Methods

### Procedure

The data were collected from July 1st to August 30th, 2018, which was the peak period of summer vacation. A two-step sampling procedure was used to obtain a representative sample of adults who had slept outside of their homes. First, we randomly selected 28 hotels in Guangdong Province. We did not consider the quality of the hotel, since recent research has found that there was no difference in sleep quality between upscale hotels and mid-scale hotels (Chen et al., 2018). Second, with the consent of the hotel staff, we visited the 28 hotels one by one. We arrived at these hotels between 9 and 12 o’clock in the morning (which is the checkout time for most customers) and randomly invited customers who had stayed there overnight and were waiting to check out in the hotel lobby. If they consented to participate, the participants followed an online link to an electronic version of the questionnaire, which was completed immediately. At each hotel, sampling was stopped after obtaining 20 samples. We invited a total of 560 customers to participate, of whom 532 customers successfully submitted a completed questionnaire. Notably, to exclude the effects of jet lag, we excluded foreign tourists and confirmed that the sample does not incorporate travel behavior across time zones. Moreover, participants with health problems or who had recently experienced a traumatic event were excluded.

Informed consent was obtained from each participant, and the study protocol was reviewed and approved by the ethical committee of the Human Research Ethics Committee of South China Normal University.

In total, 12 guests were excluded because of response bias (2.6%), and 15 guests were excluded due to completing the questionnaire in less than 1 min (2.8%). Therefore, the final sample size was 505.

### Sample

Of the 505 participants, 40.6 and 59.4% were male and female, respectively. The majority of the sample was younger than 45 years old; 42.6% were younger than 26 years old, 45.3% were between 26 and 45 years old, and 5.6% were older than 45 years old. Most of the participants were students (29.3%) and enterprise staff (26.9%). Of the participants, most had college- or university-level education (53.9%). Participants’ personal monthly income was mainly between 2,000 and 10,000 Yuan (51%). The sample included travelers who were traveling for business or leisure purposes. The average duration of stay in an unfamiliar environment was 2.572 ± 0.816 days. The demographic profiles of the participants are listed in Table 1.

**TABLE 1:** Demographic profile of participants (*n* = 505).

**Variables**	**Frequency**	**Percent (%)**	**Variables**	**Frequency**	**Percent (%)**
Age			**Occupation**		
<25	248	49.1	Government officials	97	19.2
26–45	229	45.3	Enterprise staff	136	26.9
≥46	28	5.6	Private owners	78	15.4
**Gender**			Students and others	194	38.4
Male	205	40.6	**Income (Yuan/month)**		
Female	300	59.4	<2,000	126	25.0
**Education (year)**			2,001–5,000	104	20.6
<12	141	27.9	5,001–10,000	154	30.5
13–16	272	53.9	≥10,001	121	24.0
>16	92	18.2			
**Variables**		*M ± sd*			
**Average days of stay (day)**		2.572 ± 0.816			

### Measures

#### Demographic Characteristics

Demographic characteristics were collected using a demographic questionnaire. The demographic information collected included gender, age, occupation, education, and personal monthly income.

#### Nocturnal Sleep Duration

Sleep duration is strongly correlated with severity of insomnia (Fan et al., 2018). Nocturnal sleep duration was assessed by adapting the item designed by Liu et al. (2000): ‘How many hours did you sleep at night when you stayed in this hotel?’ The aim of the questionnaire is to measure nocturnal sleep duration in a hotel.

From the responses given to this question, the sample was divided into a short sleep duration group (SSD; sleep duration < 6 h) and a normal sleep duration group (NSD; sleep duration ≥ 6 h).

#### Insomnia

Two types of insomnia were identified: insomnia in participants’ daily lives and insomnia during the trip. Three question items assessed insomnia in daily life, asking about difficulty initiating sleep (DIS), difficulty maintaining sleep (DMS), and early-morning awakening (EMA) during the past month (Liu et al., 2000). Each item was answered with a four-point scale (1 = no, 2 = less than once a week, 3 = once or twice a week, 4 = three or more times a week). If participants answered at least one of the three questions with “Less than once a week,” “Once or twice a week,” or “Three or more times a week,” they were considered as having “insomnia” (Liu et al., 2000). These three items had an acceptable reliability of 0.70 (Liu et al., 2000) with a Cronbach’s α of 0.81 in this study. The items for assessing insomnia during the trip were essentially the same as those used to measure insomnia in daily life, but the scale was modified (1 = never, 2 = rarely, 3 = sometimes, 4 = very often or always). In the current study, Cronbach’s α for these items was 0.87.

#### Sleep Habits

Individual sleep habits have a significant impact on sleep state and insomnia symptoms. Sleep habits or patterns can be classified into morning-type, evening-type, and neither-type (Adan and Almirall, 1991), and these three sleep types significantly predict all dependent variables in sleep research (Vollmer et al., 2017). One simpler subjective approach to assessing the circadian typology is the Morningness–Eveningness Questionnaire (MEQ) by Horne and Ostberg (Roveda et al., 2017), and its reduced version (rMEQ), based on 5 rather than 19 items (Adan and Almirall, 1991), has been proven to be a very good measure of circadian typology (Montaruli et al., 2017). It comprises five items, including automatic wake-up time, tired state on waking up in the morning, best time to fall asleep, “Feeling best” peak time, and a self-classification of morningness/eveningness type. The five items have a high level of internal consistency with *p* < 0.0001 (Adan and Almirall, 1991) and acceptable reliability in the Chinese population (Cronbach’s α = 0.701–0.738; Zhang et al., 2006). In the current study, Cronbach’s α was 0.80.

#### Travel Characteristics

We measured the following travel characteristics of tourists: frequency of travel, travel purpose, and mood while traveling. The first variable, frequency of travel, is a significant predictor of business tourists’ perceived satisfaction with the quality of sleep that they have in hotels (Chen et al., 2018). We used the frequency of travel in the past year to identify participants’ travel experience. The purpose of travel variable could be defined as either business travel or leisure travel. To measure mood while traveling, we adopted the Positive and Negative Affect Schedule (PANAS) of Watson et al. (1988). The PANAS measures emotions in two dimensions: 10 positive emotion attributes (interested, excited, strong, enthusiastic, proud, alert, inspired, determined, attentive, and active) and 10 negative emotion attributes (distressed, upset, guilty, scared, hostile, irritable, ashamed, nervous, jittery, and afraid). All scale items were rated from 1 (‘very slightly or not at all’) to 5 (‘extremely’). The PANAS has been used to measure emotions effectively in the Chinese population, achieving a Cronbach’s α of 0.85 and 0.83 for positive and negative dimensions, respectively (Huang et al., 2003). In the current study, Cronbach’s α for PANAS positive affect was 0.897, and for PANAS negative affect was 0.899.

#### Satisfaction With Accommodation Facilities

To estimate customer satisfaction with the hotel where they stayed overnight, this study used the scale item “I feel satisfied by hotel’s overall performance” from the H-SCI questionnaire (Deng et al., 2013). Responses were made on a scale from 1 to 5, where 1 indicated ‘strongly disagree’ and 5 indicated ‘strongly agree’. Additionally, we included three other items to measure customer loyalty; these items were taken from the ASCI questionnaire. The three items were: (1) if possible, I will revisit the hotel, (2) I tend toward praising the hotel, and (3) I would like to recommend this hotel to others (Deng et al., 2013). Responses for the four items were summed to create a total score, which was an indicator of tourists’ satisfaction with the hotel (a proxy for how satisfied they were with their sleep in the hotel). This total score was used in subsequent analyses. For the current study, Cronbach’s α was 0.913.

#### Sensitivity to the Sleep Environment

Assessment of an individual’s sensitivity to the sleep environment in hotels was adapted from items used by Pallesen et al. (2016), Chen et al. (2018), and Mao et al. (2018). The adapted scale comprised 12 items, which asked about noise insulation effectiveness, ventilation, bedding (mattress, pillow, etc.), room temperature, light, sleeping state of people in the same room, hotel service level, distance from airport or highway, hotel facilities, color scheme of the room, neighborhood environment, and green space. Participants were asked to indicate whether the hotel attributes influenced the quality of their sleep while staying at the hotel; all responses were made on a scale from 1 (did not bother me at all) to 5 (extremely bothersome). Item scores were tallied to create an overall total score, and higher scores indicated higher sensitivity to the sleep environment. For the current study, the items achieved Cronbach’s α of 0.89.

### Statistical Analysis

All data were analyzed by SPSS 23.0. We analyzed the data in two steps. First, we used a paired sample *t*-test and the paired sample *z*-test to analyze the overall impact of environmental changes on the dependent variables sleep health, which included sleep duration, perceived sleep quality, and insomnia symptoms. Following this, we divided the sample into four subgroups depending on their insomnia symptoms and the presence/absence of insomnia at home and traveling. Analyses were conducted on the basis of this group differentiation.

Adjusted chi-square tests were used to investigate the association between the development of insomnia symptoms at home and in the hotel between groups of participants who differed according to their demographic characteristics, individual sleep habits, and travel attributes. *F*-tests were used to confirm the correlation between the trajectories of insomnia symptoms from home to hotel and individual sensitivity to the sleep environment, emotions in the trip, and satisfaction with hotel service.

In subsequent analysis, binary logistic regression was used to explore the risk factors and protective factors of recovery from home insomnia and developing travel insomnia. Variables that were significant in the *F*-test and Chi-square tests at a level of *p* < 0.1 were included in the binary logistic regression model. Results from the binary logistic regression are reported as the odds ratio (OR), *p*-value, and 95% confidence interval (CI). The binary logistic regression model included all variables that retain significance after adjustment. A significance level of *p* < 0.05 was used.

## Results

### The Overall Impact of Environmental Change on Sleep Health

A paired sample *t*-test was used to determine whether participants’ sleep duration differed between home and the hotel, and the difference was significant, *t*(505) = 3.691, *p* < 0.001, Cohen’s *d* = 0.22. Participants slept for a shorter time in the hotels (6.813 ± 1.293 h) than at home (7.062 ± 1.261 h), a decrease of 0.249 h (95% CI: 0.116–0.381 h).

To investigate whether there were any differences in insomnia symptoms and perceived sleep quality at home and in hotels, we used a paired sample *z*-test. The results showed that the insomnia detection rate of the participants after staying overnight in a hotel was lower than the corresponding data for when they were at home (15.4% vs. 24.2%). The detection rate decreased by 8.8%, and this difference was statistically significant, *z* = 4.017, *p* < 0.001. Similarly, the difference in perceived sleep quality between the participants’ sleep in the hotels and sleep at home was also statistically significant (as shown in Table 2).

**TABLE 2:** Paired-samples *t/Z* test for sleep health at home and in hotel (*n* = 505).

	**Home**	**Travel**	***t/Z***
**Sleep state**			
No insomnia(frequency and percent)	383 (75.8%)	472 (84.6%)	4.017***
Insomnia (frequency and percent)	122 (24.2%)	78 (15.4%)	
**Perceived sleep quality**			
Very good (frequency and percent)	138 (27.3%)	96 (19.0%)	
Good (frequency and percent)	240 (47.5%)	294 (58.2%)	−2.319*
Poor (frequency and percent)	103 (20.4%)	100 (19.8%)	
Very poor (frequency and percent)	24 (4.8%)	15 (3.0%)	
**Sleep duration** (*M* ± *SD*) (hours)	7.063 ± 1.261	6.812 ± 1.293	3.691***

*^∗^p < 0.05, ^∗∗∗^p < 0.001.*

### Group Differences in the Effects of Environmental Changes on Sleep Health

Participants were divided into four groups based on whether they experienced symptoms of insomnia at home or during the trip. Of the 505 participants, 68.3% reported no insomnia symptoms at home and at the hotel, and were labeled the no-insomnia group (G1). The travel-insomnia group (G2) did not report insomnia symptoms at home but experienced insomnia symptoms during the stay at the hotel; 7.5% of the sample comprised this group. Approximately 16.2% reported suffering from insomnia at home but did not experience insomnia symptoms while sleeping at the hotel; these formed the recovery-from-home-insomnia group (G3). The final group consisted of participants who reported insomnia symptoms during sleeping both at home and at the hotel; this group was labeled the pan-insomnia group (G4) and comprised 7.9% of the sample.

### Analysis of Correlates of Travelers’ Sleep Health at Home and During Travel

The no insomnia group (G1), travel insomnia group (G2), recovery from home insomnia group (G3), and pan insomnia group (G4) were split by demographic characteristics for the next analysis. Table 3 lists the differences between the four groups with regards to travel characteristics, sleep habits, sensitivity to the sleep environment, positive/negative emotion, and demographics. As shown in Table 3, there were no significant between-group differences in days of stay, positive emotions, or demographics (except occupation), suggesting that these attributes were unrelated to sleep health for the four groups. However, there were significant between-group differences in travel purpose, travel frequency, sleep duration in a novel place, perceived sleep quality at home, sleep habits, sleep duration at home, environmental sensitivity of sleep, satisfaction in the hotel, and negative emotion, suggesting that these attributes were related to sleep health across the four groups.

**TABLE 3:** Demographics and other characteristics between four groups (*n* = 505).

	**G1**	**G2**	**G3**	**G4**	***F/χ^2^***
	**Frequency**				
**Year**					
≤25	172	20	36	20	1.17
>25	173	18	46	20	
**Gender**					
Male	144	13	35	13	2.07
Female	201	25	47	27	
**Occupation**					
Government officials	70	14	9	4	67.16***
Enterprise staff and private owners	141	11	44	18	
Students and others	134	13	29	18	
**Education (year)**					
≤12	100	4	24	13	8.57
13–16	187	25	39	21	
>16	58	9	19	6	
**Personal income per month (yuan)**					9.95
≤5,000	154	18	40	18	
5,001–10,000	114	19	15	14	
≥10,001	77	10	27	8	
**Travel purpose**					
Business trip	60	26	15	15	56.22***
Leisure trip	285	12	67	25	
**Travel frequency**					
Once or twice a year	190	16	39	26	25.37***
Quarterly to monthly	114	19	19	8	
Once a month or more	41	3	24	6	
**Days of stay (day)**					
1	30	7	4	3	9.74
2–6	242	26	54	30	
>7	73	5	24	7	
**Sleep habits**					
Morning type	23	8	5	8	85.99***
Neither type	25	2	34	6	
Evening type	297	28	43	26	
Duration of sleep (*M* ± *SD*) (hour)	6.96 ± 1.18	6.05 ± 1.34	7.01 ± 1.24	6.18 ± 1.38	10.81***
Sleep sensitivity to environment (*M* ± *SD*)	39.75 ± 9.57	48.58 ± 4.42	40.22 ± 8.63	44.22 ± 8.09	13.01***
Satisfaction for hotel (*M* ± SD)	14.16 ± 3.17	14.18 ± 2.87	16.84 ± 2.17	13.90 ± 3.65	25.26***
Negative emotion (*M* ± *SD*)	19.60 ± 6.75	20.42 ± 6.95	22.10 ± 6.37	24.50 ± 9.07	7.86***
Positive emotion (*M* ± *SD*)	32.11 ± 7.36	31.16 ± 8.05	33.60 ± 6.69	33.53 ± 6.76	1.62

*^∗∗∗^p < 0.001.*

To predict the risk factors and protective factors for sleep health during environmental change, we used a binary logistic regression analysis. First, since our main interest was to explore the factors associated with increased likelihood of developing travel-related insomnia and recovery from home insomnia under the influence of environmental change, we first set the no-insomnia group (G1) as the reference group and compared it in a pairwise fashion to the travel-insomnia group (G2). We then set the pan-insomnia group (G4) as the reference group and compared it with the recovery-from-home group (G3). Secondly, we entered all of the above-noted significant variables into a binary logistic regression. Please note that although there were no significant between-group differences in days of stay, we still introduce this factor into the binary logistic regression to verify the lasting effect of the FNE.

**FIGURE 1:**
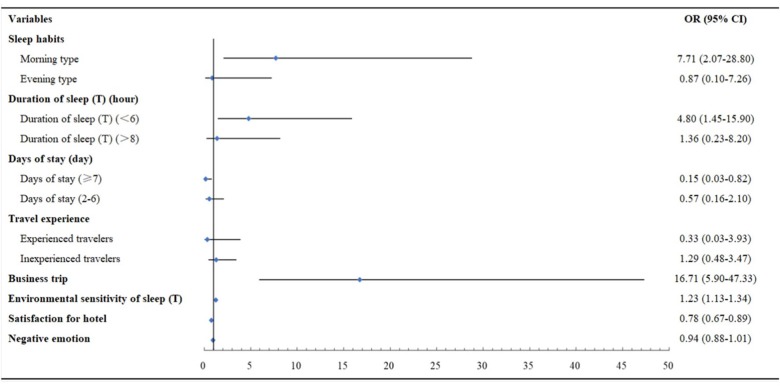
Risk and protective factors of travel insomnia groups. Participants traveling once a month or more were identified as experienced travelers; participants traveling once or twice a year were identified as inexperienced travelers; participants traveling quarterly to monthly was identified as neither type.

As shown in Figure 1, the strongest correlation was found between business travelers and the occurrence of travel insomnia in hotels, with an odds ratio of 16.99, 5% CI [5.90–47.33], followed by the correlation between being morning type and the occurrence of travel insomnia in the hotel, OR = 7.71, 95% CI [2.07–28.80]. The risk of travel insomnia tended to increase with shorter sleep duration during a change of sleep environment, OR 4.80, 95% CI [1.45–15.90], and increasing sensitivity to the sleep environment, OR = 1.23, 95% CI [1.13–1.34]. In contrast, developing travel insomnia during environment conversion was less likely for participants with high hotel satisfaction, OR = 0.78, 95% CI [0.67–0.89], and participants staying 7 days or more, OR = 0.15, 95% CI [0.03–0.82]. In the binary logistic regression of model 1, the influence of negative emotion and travel experience on travel insomnia was not significant (*p* > 0.05).

**FIGURE 2:**
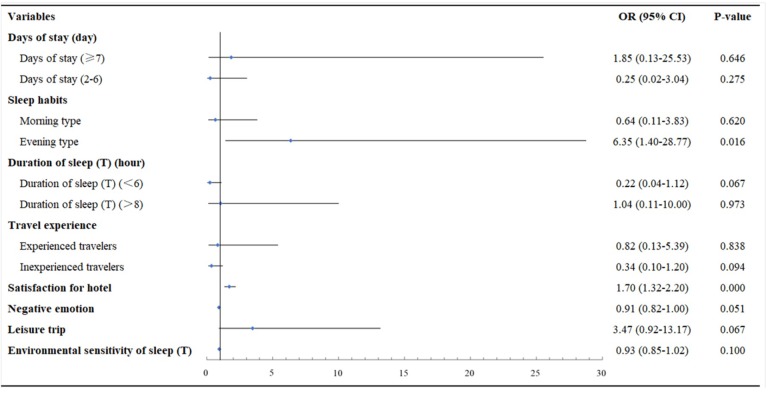
Risk and protective factors of home insomnia groups. Participants traveling once a month or more was identified as experienced travelers; participants traveling once or twice a year was identified as inexperienced travelers; participants traveling quarterly to monthly was identified as neither type.

As shown in Figure 2, the likelihood of recovering from home insomnia in unfamiliar sleep surroundings increases if the participant’s sleep habits are evening type, OR = 6.35, 95% CI [1.40–28.77], and if participants report a higher satisfaction level with the hotel, OR = 1.70; 95% CI [1.32–2.20]. Like model 1, the impact of negative emotion on the likelihood of recovering from home insomnia in the impact of environmental transformation was not significant in model 2 (*p* > 0.05). Furthermore, no significant correlation was found between the likelihood of recovering from home insomnia and travel purpose, length of stay, travel experience, sleep duration, and the environmental sensitivity of sleep.

## Discussion

To our knowledge, the current study is the first that has investigated the heterogeneous trajectories and predictors of sleep health under the impact of environmental change among tourists. Without relying on an artificial sleep laboratory environment, this study adopted a more ecologically valid approach by approaching tourists who had stayed overnight in hotels. Our findings revealed four trajectories of insomnia symptoms under the conditions of environmental changes; that is, participants could be classified as no-insomnia, travel-insomnia, recovery-from-home insomnia, and pan-insomnia. For individuals who developed travel insomnia, the risk factors included being of morning type, having short sleep duration, having sensitivity to the sleep environment, and business stress; however, satisfaction with the hotel and staying at the hotel for at least 7 days were protective factors. For the recovery-from-home insomnia group, being of evening type was a significant predictor of their symptoms, and satisfaction with the hotel was one of the important risk factors; no protective factor could be confirmed, however.

The incidence of insomnia in adults is estimated at between 8 and 20 percent (Adams et al., 2017); in the current research, however, 24.16% of the sample had insomnia. Furthermore, there were 15.47% participants who suffered insomnia symptoms when their sleep environment changed. These data indicate that in an unfamiliar sleep environment, some people will suffer from acute insomnia because of the FNE and its last continuous impact (Le Bon et al., 2001), but the FNE may be restricted to a period of 7 days or less, since staying in a hotel for over 7 days was a protective factor against suffering travel insomnia in the current study, which can be called the seven-night effect (SNE).

The prevalence of travel insomnia in our sample was 7.52%, and 16.24% of insomniacs reported that they slept better after their sleeping environment changed. This result was consistent with the view that many insomniacs are expected to sleep better in novel places than at home (Hauri and Fisher, 1986; Pallesen et al., 2016). It is worth noting, however, that leisure travelers accounted for 77% of the total participants in this study, and it may be that tourist experiences act as stress relievers (Chen et al., 2014) and may aid the underlying psychological experiences associated with recovery from bad sleep.

Interestingly, business travel is a risk factor for travel insomnia, suggesting that this kind of traveling does not act as a stress reliever (as it would for tourists). This hypothesis confirms previous findings where business travelers experience more sleeplessness in hotels than leisure travelers (Mao et al., 2018; Rundle et al., 2018). Therefore, for business people, the stress caused by business activities may be a more important trigger factor affecting sleep health than the sleep environment. However, the research also shows that there is no direct correlation between a leisure trip and experiencing better sleep in an unfamiliar place than at home. A reasonable inference from this is that, in a new sleep environment, the stress that results from the purpose of the trip is an essential risk factor for travel-related sleep disturbance; however, in the absence of this stress, the impact of environmental changes on sleep health is highlighted.

We also reported that participants who were morning types were more likely to suffer from travel insomnia but individuals who were evening types were more likely to recover from home insomnia in unfamiliar sleep surroundings. This finding differs from previous sleep studies, which found that evening types were more likely to experience a poor quality of sleep and increased severity of insomnia (Selvi et al., 2010). In the current study, all of the demographic attributes except for occupation had no effect on sleep health during sleep environment change, which demonstrates that morning/evening type is a steady, unchanging state that might be illustrated by endogenous variables (Paine et al., 2006). In fact, some types of insomnia might be caused exclusively by the maladaptive learning of poor sleep habits. Some research has shown that when the sleep-wake cycle is interrupted during travel, morning-type people and evening-type people perform differently and self-adjust differently. For example, morning-type people tend to have regular social rhythms (Ong et al., 2007), they always get up early and at a regular time, and have good sleep (Byrne et al., 2018); however, their sleep is easily interrupted, and they are vulnerable to the effects of jet lag and do not adapt easily to a novel sleeping environment (Ong et al., 2007) or to the disruption of their biological clock (Flower et al., 2003). In contrast, evening-type people have more irregular sleep rhythms, but they display more plasticity in adjusting their sleep duration because they have flexible sleep-wake habits. Furthermore, after engaging in a variety of travel activities during the day, evening-type people will reach the peak of their circadian rhythm earlier than they would in their normal environment and appear to consciously increase the amount of time that they sleep at night when in a hotel so that they can meet their greater need of sleep (Ong et al., 2007). Hence, being a morning-type person is a risk factor for developing travel insomnia, while being an evening-type person is a risk factor for developing a recovery pattern in a novel sleep environment.

Additionally, short sleep duration is a risk factor of travel insomnia, which is in accord with Fan et al. (2018). Specifically, people who sleep for short periods are more likely to suffer from insomnia regardless of changes in their environment. One possible explanation is that our sample comprised largely young adults, who may sleep for short periods. Although some researchers consider sleep quantity or duration to be less important than sleep quality (Bassett et al., 2015; Litwiller et al., 2017), our study suggests that sleep duration is an important factor that has a significant effect on sleep health in novel environments.

Our results also show that individuals are more likely to suffer travel insomnia if they have higher sensitivity to the sleep environment, which is in line with findings reported by Pallesen et al. (2016) and Mao et al. (2018). The individual’s sleep response depends largely on how sensitive they are to their sleep environment. Therefore, individuals who are sensitive to the sleep environment are more likely to develop travel insomnia; this finding supports the explanation of the FNE (Thomas et al., 2005; Ellis et al., 2012; Ellis et al., 2012).

A final notable finding is that travelers are less likely to develop travel insomnia symptoms and more likely to recover from home insomnia in a strange environment when they report higher satisfaction rather than low satisfaction with the hotel. This finding strongly suggests that the relationship between sleep health and hotel customer satisfaction is bi-directional, though past studies have mainly focused on how sleep quality affects hotel satisfaction (Schuckert et al., 2015). Therefore, we can cautiously infer that satisfaction with the accommodation environment protects individuals’ sleep health during an environment transformation.

### Implications

The FNE is a well-known phenomenon in sleep research and frequently occurs in research conducted in sleep laboratories (Le Bon et al., 2001). The current study tested the FNE in a more ecologically valid environment, and our findings not only confirmed the existence and duration limit of the FNE outside of the laboratory but also provided further insight on how the FNE was affected by behavioral factors.

In modern society, sleep is no longer a private activity that occurs only at home, and people’s sleep environment may change regularly. The current paper explores the sleep health problems that occur when individuals are exposed to a new sleep environment while traveling. Although a great deal of research has focused on persistent/chronic insomnia, there is a dearth of research investigating acute insomnia, especially the transition from normal sleep to acute insomnia (Ellis et al., 2012). In our study, we investigated acute insomnia caused by sleep environment transformation in travel since this can provide an important reference for individual sleep health management plans.

Individuals, but especially travelers, should realize that good sleep does not rely entirely on the unilateral effort of accommodation providers; instead, individuals should be made aware that their sleep habits may impact their sleep experience while traveling; for instance, morning people should ensure consistency with the sleeping patterns that they have at home or engage in exercise to avoid the risk of sleeplessness. Additionally, travelers could either ask hotel employees to provide them with the necessary resources/equipment/furniture to ensure good quality sleep or equip themselves with sleep supplies. Importantly, business travelers should try to keep a balance between work and relaxation to avoid developing travel insomnia, and leisure travelers should pay more attention to their sleep habits to maintain their physical and mental health (Kumar et al., 2014).

Moreover, the current research has some interdisciplinary implications, and its conclusions can provide practical guidance for hotels’ customer sleep management. At the present time, most hotels do not take the initiative to specifically improve guests’ sleep quality, although hotels are aware of the importance of sleep with regards to visitors’ hotel satisfaction ratings (Pallesen et al., 2016; Mao et al., 2018).

The findings of the current manuscript underline the importance of reducing the risk factors and strengthening the protective factors of travel insomnia, as well as enhancing the factors that assist with recovery from home insomnia. First, hotel characteristics have a positive relationship with sleep experience, which is evident among participants with high sleeping environmental sensitivity and high hotel satisfaction. For these reasons, hotels need to provide a comfortable sleeping environment and improve service quality to guarantee guests’ satisfaction. For example, My City Hotel offers a “Good Night Sleep Package” for guests, which includes a quiet courtyard side room, lavender sachet, natural light wake-up, calming herbal tea, and silent housekeeping^1^. Second, personal sleep and travel characteristics, such as circadian typology, sleep duration, and trip purpose, are significantly associated with sleep health while traveling. It is the responsibility of hotels to assist travelers in recognizing their own circadian rhythm. For example, hotel employees could arrange a customized morning call or midnight service from the executive lounge to business travelers with an eveningness preference. Furthermore, upon check-in, tourists’ circadian typology and sleep habits could be measured by a sleep specialist, and the hotel could offer a personalized sleep service to guests based on the results. There are examples of such services in practice: Canyon Ranch offers tourists a specialized sleep medicine program^2^ that includes the diagnosis and treatment of sleep disorders, sleep screening test, and polysomnography by physicians or sleep specialists, and Six Senses hotels^3^ have also launched a personalized sleep program where tourists are given a sleep tracker and the results are explained by a specialist. Finally, hotels should follow the recent trend where high-tech products are used to improve guests’ sleep. For example, together with Lark Technologies, Westin hotels provide sleep monitors, silent alarm clocks, and personal sleep coaches. With the development of virtual reality and augmented reality or similar technologies, in the near future, hotels may be able to enhance rooms with the use of digital soundproofing, auto-massage de-stress pillows, and virtual lighting selection.

### Limitation and Future Research

There are several limitations to our study. First, the data collected were self-reported via online surveys. Although the self-reported questionnaire has a clear structure and objective score, some participants may provide incorrect information. Furthermore, their responses may be vulnerable to forgetfulness. In future research, researchers should employ experimental approaches to evaluate sleep quality objectively (e.g., actigraphy and polysomnography; Mao et al., 2018). Secondly, the findings in the present study were derived from a relatively small sample. Future research should aim to recruit a large sample with a particular focus on business travelers. The focus of the current study was on the home-hotel environment change, but it is possible that we would have seen different results had we considered the home-hotel-home environment changes. In other words, transient insomnia is often exhibited as impaired functioning and alertness the following day (Thomas et al., 2005), and these symptoms may be present among travelers; however, the manner in which the sleep environment change affects sleep-wake patterns after travelers have returned to a familiar sleep environment is largely unknown. This question is vital in creating effective measures to ensure the health of travelers who are exposed to a strange environment. To answer this question, we need long-term monitoring of sleep-wake patterns after returning to the home environment that employs effective methods (e.g., a daily sleep log). Also, we did not measure other variables in our study because the factors that influence individuals’ sleep in a new environment are complicated. For example, we considered the effects of daily sleep habits on the sleep health of the subjects in the new environment but did not inspect the sleep rhythm of the participants in hotels, such as their sleep timing. If we could collect these data, more specific suggestions for different groups might be proposed. We analyzed the impact of travel purpose on participants’ sleep health in unfamiliar sleeping surroundings but did not pay close attention to some specific situations for business travelers. Business travelers might have jetlag, experience work stress due to business activities, and not be able to sleep at an appropriate sleep time due to working in a new place, etc. Finally, there might be interaction between some factors; for instance, a higher level of noise at night might be a cause of lower hotel satisfaction and higher environmental sensitivity in a new sleep location; the association of morningness people with worse sleep quality in hotels could also be explained by the effect of noise around the hotel or higher noise sensitivity in a new sleep location. Therefore, we suggest that subsequent research scholars predict other relevant information, such as social background, physical and mental health status, and specific travel characteristics, including travelers who crossed time zones, the distance of travel, different work activities when traveling for business, travel hours, and different forms of transportation, so that they can add further insight into acute travel-related insomnia.

## Data Availability Statement

The datasets presented in this article are not readily available because they are being used in ongoing research. Requests to access the datasets should be directed to the corresponding author.

## Ethics Statement

The studies involving human participants were reviewed and approved by the ethical committee of the Human Research Ethics Committee of South China Normal University. The patients/participants provided their written informed consent to participate in this study.

## Author Contributions

WX undertook the data analyses, conducted the literature searches and analyses, and wrote the manuscript. FF conceptualized and supervised the whole study. HQ undertook the data analyses. All authors contributed to and have given approval to the final manuscript.

## Conflict of Interest

The authors declare that the research was conducted in the absence of any commercial or financial relationships that could be construed as a potential conflict of interest.

## Footnotes

1 MyCityHotel (2018). Good night’s sleep guarantee. Retrieved from https://www.mycityhotel.ee/

2 CanyonRanch (2018). Explore Experience-Medical. Retrieved from https://www.canyonranch.com/tucson/

3 SixSenses (2018). Sleep With Six Senses. Retrieved from https://www.sixsenses.com/wellness/
